# Adsorption Behavior of the L-Theanine onto Cation Exchange Resin ZGSPC106Na and D001SD

**DOI:** 10.3390/foods11223625

**Published:** 2022-11-13

**Authors:** Yusi Yang, Zhanbo Dong, Yongkang Wang, Fengyi Xiao, Jinliang Yang, Dong Zhao, Jianhui Ye, Xinqiang Zheng, Yuerong Liang, Jianliang Lu

**Affiliations:** 1 Tea Research Institute, College of Agriculture and Biotechnology, Zhejiang University, Hangzhou 310058, China; 2 Pingyang Agricultural and Rural Bureau of Zhejiang Province, Wenzhou 325405, China; 3 Wulongtan Tea Industry Co., Ltd. of Ningbo City, Ningbo 315166, China

**Keywords:** separation of tea components, amino aicd, decaffeination, adsorption capacity, adsorption selectivity, adsorbent

## Abstract

Adsorption is an important technology for the separation of different tea components. The adsorption behavior of L-theanine onto adsorbents was comprehensively studied in this paper. Among tested adsorbents, cation exchange resin ZGSPC106Na and D001SD were suitable for separating L-theanine, PVPP and PA-6 for catechins and macroporous resin HPD-400 for caffeine. Adsorption of L-theanine onto the cation resins was significantly influenced by the acidity, contact time and temperature. The adsorption behavior could be described by the pseudo-second-order rate equation and fitted to Langmuir and Freundlich models. ZGSPC106Na exhibited higher adsorption capacity, while D001SD showed higher adsorption selectivity. These might be attributed to the distinctive structure of the two resins and different ionization of the adsorbates. A method for simultaneous preparation of decaffeinated polyphenols, caffeine-enriched extract and decaffeinated L-theanine was established through successive separation on the columns fulfilled with PA-6, HPD-400 and D001SD, respectively.

## 1. Introduction

Tea is a non-alcohol beverage commonly consumed worldwide and possesses many bioactive components, including L-theanine, catechins, caffeine and polysaccharides. L-theanine is a nonproteinic amino acid and has exclusively been isolated from the genus *Camellia* and a kind of mushroom, *Xerocomus badius* [1]. It can be biosynthesized in many species or varieties of *Camellia* plants, but accumulates at a higher level only in *C. sinensis* [2], accounting for 1–2% dry weight of tea leaves and 50% of the total free amino acids [3]. L-Theanine has been recognized as an umami-enhancing compound in tea infusion [4] and is known for its effect on human relaxation [5], including stress-relieving, improvement of cognitive performance, invoking sleep, anti-cancer and so on [6]. Catechins are the major components of polyphenols, accounting for 12–24% of the dry weight of tea [7] and can exert their anti-cancer effects [8] and prevent cardiovascular disease [9] mainly through their strong antioxidant activity [10] and unique immunomodulation potential [11]. Caffeine, being up to 3–5% of dry weight, can excite the central nervous system and has a refreshing effect [12]. Although these three types of compounds have their own unique biological functions, contamination of each other will inevitably lead to predictable or unpredictable side effects [13,14]. Therefore, L-theanine, catechins and caffeine should be separated and purified prior to application in pharmaceuticals and functional foods.

Many attempts have been made to isolate L-theanine from tea, since natural L-theanine obtained from plants enjoys the greatest popularity among customers in the consideration of the high safety and bioactivity of the product as well as easy accessibility of plant resources, despite the convenience and low cost of chemosynthesis and bacterial pathway biosynthesis [15,16,17]. L-theanine has been successfully isolated from tea extract through preparative HPLC and the molecularly imprinted polymer (MIP) technique [18,19]. However, these techniques are only suitable for the small-scale preparation of L-theanine, but not for large-scale production [20]. Resin adsorption is widely used for the isolation of bioactive compounds from plant materials [21,22,23,24,25] and applied in the removal of dyes from wastewater [26,27,28]. Crude L-theanine can be separated from matrixes by using cation exchange resin No. 732 as an adsorbent and the crude L-theanine could be further purified by application of preparative HPLC [19], a second No.732 column separation [21] or recrystallization [22]. However, except for No. 732, very few cationic resins have been tested to isolate the L-theanine, which limits the choice of highly efficient adsorbents in the preparation of L-theanine and the mechanism clarification of adsorption behaviors. 

The isolation of L-theanine from tea is faced with some difficulties due to the presence of high levels of polyphenols and caffeine. In this study, the adsorption behaviors of L-theanine by cation exchange resins ZGSPC106Na and D001SD were comprehensively studied after the adsorption selectivity of different adsorbents had been tested, and a method was also proposed to simultaneously prepare the decaffeinated catechins, caffeine-enriched extract and decaffeinated L-theanine from green tea by successive column separation.

## 2. Materials and Methods 

### 2.1. Chemicals and Plant Materials

Crosslinked polyvinylpyrrolidone (PVPP) was obtained from Sigma (Sigma-Aldrich, St. Louis, MO, USA). Polyamide PA-6 was purchased from Sijia Biochemical Plastic Factory (Taizhou, China). HPD-400 macro-porous resin and D001SD cation exchange resin were purchased from Shandong Lukanglike Pharmaceutical Co., Ltd. (Jining, China) and cation exchange resin ZGSPC106Na was purchased from Zhejiang Zhengguang Industrial Co., Ltd. (Hangzhou, China). Caffeine and catechins standards, including epigallocatechin gallate (EGCG), epigallocatechin (EGC), epicatechin gallate (ECG), epicatechin (EC), gallo-catechin gallate (GCG), gallo-catechin (GC), catechin gallate (CG) and (+)-catechin (C), were supplied by Sigma (Sigma-Aldrich, St. Louis, MO, USA). L-theanine standard (99.9%) and a tea extract with a high level of L-theanine were supplied by Gosun Biotechnologies Co., Ltd. (Hangzhou, China). The latter was composed of 30.76% L-theanine, 18.82% catechins, 5.10% caffeine and 12.24% polysaccharides. UV derivatizer 1-Fluoro-2,4-dinitrobenzene (FDBN) was obtained from Tokyo Chemical Industry (Tokyo, Japan). Ninhydrin hydrate was purchased from Aladdin Co. (Shanghai, China). Folin & Ciocalteu′s phenol reagent and gallic acid were products of Sigma-Aldrich (Shanghai, China). Acetonitrile and methanol of HPLC grade were purchased from Tedia Company Inc. (Fairfield, OH, USA). All other reagents were of analytical grade. The water used in the experiments was purified by the EASYPure II UV-Ultra-Pure Water System (Barnstead International, Dubuque, IA, USA). Green tea was made according to a common protocol; in detail, the tender shoots, with one bud and two leaves, were plucked from cultivar ‘Fudingdabai’ and withered at room temperature for 8 h to release some moisture and grassy odor, then the withered shoots were fixed in a mini microwave fresh tea leaves fixing machine at 800 w for 2 min (Guangzhou Zhiya Industrial Microwave Equipment Co., Ltd., Guangdong, China) to denature the enzymes. The fixed shoots were rolled in a model 25 rolling machine (Zhejiang Shangyang Machinery Co., Ltd., Quzhou, China) for 15 min and then dried at 120 °C for 15 min and at 80 °C until completely dry (usually for 4 h) in a 6CTH-3.0 tea drier (Zhejiang Shangyang Machinery Co., Ltd.).

### 2.2. Pretreatment of the Adsorbents

All of the adsorbents were first drenched in highly purified water for 6 h and washed several times until the eluate was colorless. Cation exchange resins were then soaked in 4% *w*/*v* NaOH, the volume of which was 3 times that of the swollen resin, for 24 h, after which it was washed with water to pH 7. Then the resins were soaked in 5% *v*/*v* HCl, the volume of which was 3 times that of the swollen resin, for 24 h and washed with water to pH 7. The HPD400, PA-6 and PVPP were soaked in ethanol for 24 h and washed with ethanol until the eluate was clear, then with water, the volume of which was 5 times of the swollen resin. The HPD400, PA-6 and PVPP were soaked in 5% *v*/*v* HCl, the volume of which was 3 times that of the swollen resin, for 2–4 h and washed with water to pH 7, after which they were soaked in 2% *w*/*v* NaOH, the volume of which was 3 times that of the swollen resin, for 2–4 h, and washed with water to pH 7. Surface moisture was removed from the treated adsorbents by vacuum filtration. 

### 2.3. Screening of the Adsorbents for L-theanine Separation

To screen the adsorbents suitable for L-theanine separation, the adsorption selectivity of different adsorbents over caffeine, catechins and L-theanine was tested. Adsorbate solution (30 mg mL^−1^) was prepared by dissolving 12.000 g tea extract containing 30.76% L-theanine, 18.82% catechins, 5.10% caffeine and 12.24% polysaccharides (Gosun Biotechnologies Co., Ltd., Hangzhou, China) into 400 mL ultrapure water and centrifuged at 6000× *g* for 20 min at room temperature. The supernatant was collected and used in the following adsorption tests. The pretreated ZGSPC106Na, D001SD, PVPP, PA-6 and HPD-400 (equivalent to 0.500 g dry weight for each adsorbent) were mixed with 25.0 mL supernatant, respectively. The mixture was shaken in the HZ-9201K constant temperature oscillator (Taicang, China) at 25 °C and 150 rpm for 12 h and centrifuged to separate the supernatant and the adsorbent. The initial and final concentrations of total catechins (TCs), caffeine, L-theanine and tea polysaccharides (TPs) in the solution were analyzed before and after adsorption. The tests were carried out in triplicate.

The adsorption capacities of each adsorbent for the target compounds can be calculated by Equation (1):

(1) $$Q_{e}=\frac{V\left(C_{0}-C_{e}\right)}{M}$$

where *Q_e_* is the adsorption capacity (mg g^−1^) at equilibrium, *C*_0_ and *C_e_* are the concentrations of the adsorbate (mg mL^−1^) before and after adsorption, respectively. *V* is the solution volume (mL) and *M* is the adsorbent dry weight (g).

The selectivity coefficient $K_{A}^{B}$ quantifies the ability of an adsorbent to select one of two solutes simultaneously present in a solution. The coefficient can be calculated by Equation (2):

(2) $$K_{A}^{B}=\frac{R_{B}/C_{B}}{R_{A}/C_{A}}=\frac{R_{B}}{R_{A}}\times \frac{C_{A}}{C_{B}}$$

where *A* and *B* represent adsorbate *A* and *B*, respectively, *C_A_* and *C_B_* are the concentrations (mg mL^−1^) of adsorbate *A* and *B* in the solution at equilibrium, respectively, and *R_A_* and *R_B_* are the adsorption capacities (mg g^−1^) of adsorbate *A* and *B* by the adsorbent at equilibrium, respectively. High $K_{A}^{B}$ value represents good selectivity for adsorbate *B* over adsorbate *A* [23].

### 2.4. Optimization of Separation Parameters for L-theanine Preparation

#### 2.4.1. Different Adsorption Acidity Tests 

The model solution, containing 20 mg mL^−1^ L-theanine and 10 mg mL^−1^ caffeine, was prepared and adjusted to different acidity (pH 1–9) through an appropriate addition of 1.0 M HCl or NaOH. An amount of model solution (50 mL) was mixed with pretreated cation exchange resins (2.500 g equivalent to dry weight) and shaken at 25 °C and 150 rpm for 12 h in an HZ-9201K constant temperature oscillator (Taicang, China). The supernatant was obtained after the mixture was centrifuged at 6000× *g* and 25 °C for 10 min in a Thermo Sorvall ST16R centrifuge (Thermo Scientific Co., Rockford, IL, USA). The concentrations of L-theanine and caffeine in the supernatant were analyzed by a Shimadzu LC-20A HPLC (Shimadzu Corp., Kyoto, Japan). Test was repeated three times. The adsorption capacity and selectivity were calculated according to Equations (1) and (2).

#### 2.4.2. Different Adsorption Time Tests

100 mL model solution (pH 5.67) was treated with 5000 g (equivalent to dry weight) cation exchange resin in 250 mL Erlenmeyer flasks and shaken at 25 °C and 150 rpm. 100 µL aliquots of extraction solution were taken at different time intervals. Samples were properly diluted with ultrapure water (1:1, *v*/*v*, for L-theanine detection; 1:9, *v*/*v*, for caffeine detection) and centrifuged at 6000× *g* and 25 °C for 10 min. The concentrations of L-theanine and caffeine in the supernatant were analyzed by HPLC. Experiments were performed in triplicate.

The pseudo-first-order rate equation as well as the pseudo-second-order rate equation were employed to describe the mechanism of adsorption, which were expressed by Equations (3) and (4), respectively:

(3) $$ln(Q_{e}-Q_{t})=lnQ_{e}-k_{1}t$$

(4) $$\frac{t}{Q_{t}}=\frac{1}{k_{2}Q_{e}{}^{2}}+\frac{t}{Q_{e}}$$

where *Q_e_* and *Q_t_* are the adsorption capacities (mg g^−1^) of the adsorbate in the adsorbent at equilibrium and at time *t* (min), respectively; *k*_1_ (min^−1^) and *k*_2_ (g mg^−1^ min^−1^) are the rate constants of pseudo-first-order adsorption and pseudo-second-order adsorption, respectively. Parameter *k*_1_ and *Q_e_* can be calculated from the slope and intercept of the plot of ln (*Q_e_* − *Q_t_*) vs. t, respectively. Parameter *k*_2_ and *Q_e_* can also be obtained from the slope and intercept of the plot of *t*/*Q_t_* vs. *t*, respectively.

#### 2.4.3. Different Adsorption Temperature Tests

Pretreated adsorbent (equivalent to 0.250 g dry weight) was shaken with 5 mL L-theanine solution (10–80 mg mL^−1^) in a HZ-9201K constant temperature oscillator (Taicang, China) at 150 rpm and different temperatures (293 K, 303 K and 323 K, respectively) for 12 h. The concentrations of L-theanine in the supernatant were analyzed by HPLC.

The Langmuir and Freundlich models were used to describe the adsorption mechanism. They are expressed by Equations (5) and (6), respectively:

(5) $$1/Q_{e}=1/(Q_{m}k_{L}C_{e})+1/Q_{m}$$

(6) $$lnQ_{e}=(1/n)lnC_{e}+lnk_{F}$$

where *Q_e_* and *C_e_* are the concentrations of adsorbate in the adsorbent phase (mg g^−1^) and solution phase (mg mL^−1^) at equilibrium, respectively; *Q_m_* (mg g^−1^) is the maximum monolayer adsorption capacity; *K_L_* (mL mg^−1^) is the Langmuir affinity constant; *K_F_* (mg^(*n*−1)/*n*^ g^−1^ mL^1/*n*^) is the Freundlich constant related to adsorption capacity; and *1/n* is the constant related to heterogeneity factor. *Q_m_* and *K_L_* can be calculated from the slope and intercept of the plot of 1/*Q_e_* vs. 1/*C_e_*, while *1/n* and *K_F_* can be calculated from the slope and intercept of the plot of *lnQ_e_* vs. *lnC_e_*.

#### 2.4.4. Different Eluent Tests

Different eluents, including NaCl solution (1.0 mol L^−1^, pH = 5.90), Na_2_HPO_4_ solution (0.133 mol L^−1^, pH = 9.38), Na_2_CO_3_ solution (1.0 mol L^−1^, pH = 11.50) and ammonia solution (0.5 mol L^−1^, pH = 11.48 and 1.0 mol L^−1^, pH = 11.63), were tried to elute the adsorbates from adsorbents. An amount of pretreated adsorbent (equivalent to 0.500 g dry weight) were shaken with 10 mL L-theanine solution (20 mg mL^−1^) at 25 °C and 150 rpm for 12 h. The remaining solution and the adsorbents were separated through vacuum filtration. The filtrate was centrifuged at 6000× *g* for 10 min and the concentration of L-theanine in the supernatant was analyzed by HPLC. The adsorbents were then shaken with 20 mL of different eluents at 25 °C and 150 rpm for 12 h. Vacuum filtration was then performed, after which the filtrate was centrifuged at 6000× *g* for 10 min and the supernatant was subject to HPLC analysis.

The elution rate was calculated by Equation (7):

(7) $$E\left(\%\right)=\frac{C_{2}L_{2}}{\left(C_{0}-C_{1}\right)L_{1}}\times 100\%$$

where *C*_0_ and *C*_1_ were the concentrations (mg mL^−1^) of the adsorbate in the solution before and after adsorption, respectively and *C*_2_ was the concentration (mg mL^−1^) of the adsorbate in the eluate after elution. *L*_1_ and *L*_2_ were the volumes (mL) of the adsorption solution and eluate, respectively.

### 2.5. Simultaneous Preparation of Different Tea Components

#### 2.5.1. Preparation of Green Tea Extract 

Green tea (1000 g) was soaked in 8 L of ultrapure water at 95 °C for 20 min. Hand stirring with a glass rod was performed once every 5 min. The extract was filtered through two layers of C32 × C32–68 × 68−1 gauze (Huadong Medicine Co. Ltd., Hangzhou, China) to separate the residue, which was then extracted again as above. The filtrates were combined and cooled to room temperature and then centrifuged at 6000× *g* for 10 min. The supernatant was concentrated to 3 L by rotary vacuum evaporation at 45 °C. The concentrated extract was spray-dried and 282 g of powder was obtained. 10.0 mg powder was dissolved in 20 mL ultrapure water and the supernatant was subject to determine the content of components after the solution was centrifuged at 12,000× *g* for 10 min at 4 °C. The powder contained 281.34 mg g^−1^ catechins, 114.81 mg g^−1^ TPs, 64.53 mg g^−1^ caffeine and 32.57 mg g^−1^ L-theanine, respectively.

#### 2.5.2. Simultaneous Preparation of Active Compounds through Column Separation

Fixed bed columns were prepared through a wet package with PA-6 (400 mm × 40 mm i.d.; bed volume (BV) = 500 mL appr.), HPD-400 (350 mm × 30 mm i.d.; BV = 250 mL appr.) and D001SD (320 mm × 16 mm i.d.; BV = 65 mL appr.) and balanced with highly purified water. Green tea extract (50 g) was dissolved in 500 mL water and loaded onto the PA-6 column at a flow rate of 500 mL h^−1^ and the effluent (E_1_) was collected. The PA-6 bed was eluted with 1000 mL ultrapure water at a flow rate of 1000 mL h^−1^ and the eluate (E_2_) was collected, then eluted with 1000 mL 80% ethanol solution and the eluate (E_A_) containing polyphenols (catechins) was recovered. The E_1_ and E_2_ were pooled and vacuum-evaporated to 250 mL (E_1&2_). The E_1&2_ was then loaded onto the HPD-400 column at a rate of 250 mL h^−1^ and the effluent (E_3_) was collected. The HPD-400 bed was washed with 500 mL ultrapure water at a flow rate of 500 mL h^−1^ and the eluate (E_4_) was collected, then eluted with 500 mL 80% ethanol solution and the eluate (E_B_) containing catechins and caffeine was recovered. The E_3_ and E_4_ were mixed and evaporated to 130 mL (E_3&4_). The E_3&4_ was loaded onto the D001SD column and the effluent (E_5_) was gathered. The D001SD bed was washed with 150 mL 80% ethanol and the eluate (E_6_) was gathered. The bed was finally eluted with 150 mL 0.5 mol L^−1^ aqueous ammonia and the eluate (E_C_) containing L-theanine was recovered. The collected eluate E_A_, E_B_ and E_C_ were vacuum-evaporated to remove the ethanol, ammonia and partial water and then freeze-dried into powders (labeled as powder P_A_, P_B_ and P_C_ accordingly) in an Alpha 1–4 LD plus freeze dryer (Marin Christ, Osterode, Germany), respectively. The powders were stored in a desiccator to prevent moisture absorption. The separation was carried out in triplicate.

### 2.6. Analysis of the Target Compounds

#### 2.6.1. Detection of Catechins and Caffeine

Catechins and caffeine were detected by an LC20A HPLC (Shimadzu, Kyoto, Japan) coupled with a TC-C_18_ column (250 mm × 4.6 mm, particle size 5 μm; Agilent Technologies Inc., Santa Clara, CA, USA) after the solution was properly diluted. Analysis conditions were as follows: injection volume, 10 µL; oven temperature, 35 °C; mobile phase A, acetonitrile/acetic acid/water (6/1/193, *v*/*v*/*v*); mobile phase B, acetonitrile/acetic acid/water (60/1/139); gradient elution, linearly increasing the mobile phase B from 30% to 85% in the early 33 min and holding mobile phase B at 85% for another 5 min; flow rate, 1 mL min^−1^; detecting wavelength, 280 nm. Catechins and caffeine were qualified and quantified by comparing with retention time and peak area of the authentic standards. The detailed operation was carried out as described in the previous paper [23].

The content of polyphenols was also estimated by colorimetry in which Folin & Ciocalteu′s phenol was used as a chromogenic agent [29].

#### 2.6.2. Detection of L-theanine

The L-theanine was detected through HPLC after proper dilution and derivatization according to a previous paper [30]. For the derivatization of theanine, 100 μL sample solution was mixed with 100 μL NaHCO_3_ aqueous solution (0.5 mol L^−1^, pH 9.0) and 100 μL 1% FDBN (dissolved in acetonitrile) and incubated at 60 °C for 1 h under dark condition. The derivatized solution was cooled to room temperature and mixed with 500 μL KH_2_PO_4_ solution (10 mmol L^−1^, pH 7.0), then centrifuged at 6000× *g* for 10 min. The supernatant was used for HPLC detection. The analysis conditions were as follows: column, TC-C_18_; oven temperature, 35 °C; injection volume, 10 μL; detection wavelength, 365 nm; mobile phase A, 5 mmol L^−1^ sodium acetate/tetrahydrofuran/water (18/1/1, *v*/*v*/*v*, pH 5.7); phase B, methanol; flow rate 1.0 mL; gradient elution, 35% phase B to 41% phase B by linearly increasing in the early 9 min and then back to 35% B in the next 9 min. The theanine was qualified and quantified by comparing with retention time and peak area of the authentic standard.

The total of amino acids was estimated by the colorimetry method in which ninhydrin was used as a chromogenic agent [31].

#### 2.6.3. Analysis of TPs

The anthrone-sulfuric acid colorimetric method was employed to determine the soluble TPs in the samples, then 1 mL properly diluted sample was transferred into a glass tube which was incubated in an ice bath and precooled with 4 mL anthrone (0.1%)—sulfuric acid (80%) reagent was mixed with the sample and cooled in the ice bath. The cooled mixture was incubated at 100 °C for 5 min and then cooled in the ice water for 30 min. The absorbance was detected at 625 nm by an HP8453E UV-VIS spectrophotometer (Hewlett Packard Company, Palo Alto City, CA, USA). Meanwhile, a series of glucose standard solutions (0–0.1 mg mL^−1^) was prepared and the color development reaction was performed as above through the replacement of the sample with the same volume of standard solution. The standard curve was plotted by using the recorded absorbance and concentration of glucose as X and Y (mg mL^−1^). The concentrations of TPs in the samples were calculated according to the standard curve (Y = 0.1252X + 0.0042).

### 2.7. Statistics 

Microsoft Excel 2018 (Microsoft Corp, Seattle, WA, USA) was used for the arrangement of raw data and calculation of adsorption capacities, adsorption selectivity and coefficients as well as chart drawing, and the significance analysis was performed by SAS (Cary, NC, USA).

## 3. Results

### 3.1. Adsorbent Suitable for Separation of Different Tea Components 

**Figure 1:**
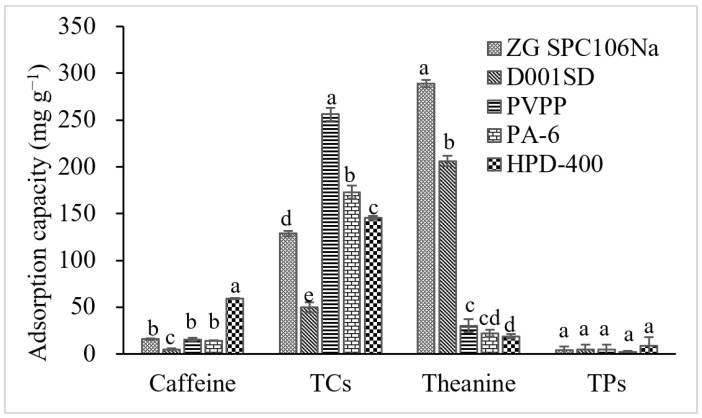
Adsorption of caffeine, catechins, L-theanine and tea polysaccharides onto the various adsorbents. TCs, total catechins; TPs, tea polysaccharides. Different letters (a–e) for each adsorbate indicated significantly different adsorption capacities between various adsorbents at *p* < 0.05.

The adsorption tests were carried out to screen the suitable adsorbent from five candidates for separating different target compounds, including L-theanine, caffeine, catechins and TPs. The results showed that the L-theanine could only be well adsorbed by ZGSPC106Na (288.79 mg g^−1^) and D001SD (206.11 mg g^−1^), the catechins could be by PVPP (256.19 mg g^−1^), PA-6 (172.82 mg g^−1^), HPD-400 (145.25 mg g^−1^) and ZGSPC106Na (128.72 mg g^−1^), the caffeine only by HPD-400 (58.85 mg g^−1^) and the TPs could not be adsorbed, among the tested adsorbents (Figure 1). According to the calculation of selectivity coefficient (*K*), ZGSPC106Na and D001SD showed higher adsorption selectivity for L-theanine instead of caffeine and catechins since the *K* values for adsorbing L-theanine vs. caffeine ($K_{Caf}^{Thea}$) and L-theanine vs. TCs ($K_{TCs}^{Thea}$) were bigger than 1.7 (Table 1); meanwhile, these two resins also possessed higher $K_{Caf}^{TCs}$ coefficients although relatively low adsorption capacities for catechins and caffeine were observed. Although higher adsorption capacities for L-theanine, TCs and caffeine were observed by ZGSPC106Na, the selectivity coefficients, $K_{TCs}^{Thea}$ and $K_{Caf}^{Thea}$ of D001SD were significantly higher than that of ZGSPC106Na (Table 1). PVPP showed the highest $K_{Caf}^{TCs}$ and $K_{Thea}^{TCs}$, followed by PA-6, while HPD-400 possessed higher $K_{Thea}^{Caf}$ and $K_{Thea}^{TCs}$. This confirmed that PVPP and PA-6 had higher adsorption selectivity for catechins and HPD-400 had higher selectivity for caffeine and catechins. Combining the adsorption capacity with selectivity, the ZGSPC106Na and D001SD were quite suitable for separating the L-theanine from a complex matrix. In addition, PVPP and PA-6 could be used as adsorbent candidates for adsorbing the catechins and HPD-400 as caffeine and catechins adsorbent, which were consistent with previous reports [23,24,32]. Considering the physical and elution characteristics, PA-6 was recommended for separating the catechins because of its good stiffness and easy elution.

### 3.2. Adsorption and Elution of L-theanine

#### 3.2.1. Effect of pH Value

**Figure 2:**
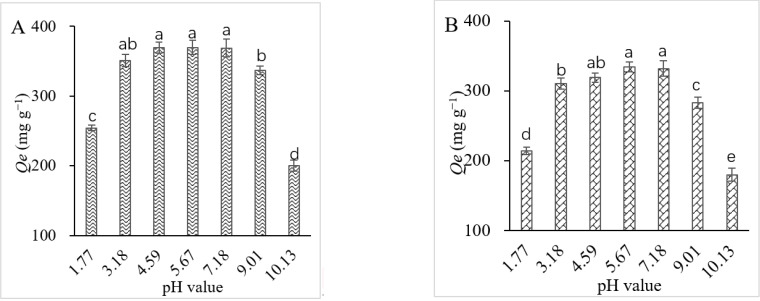

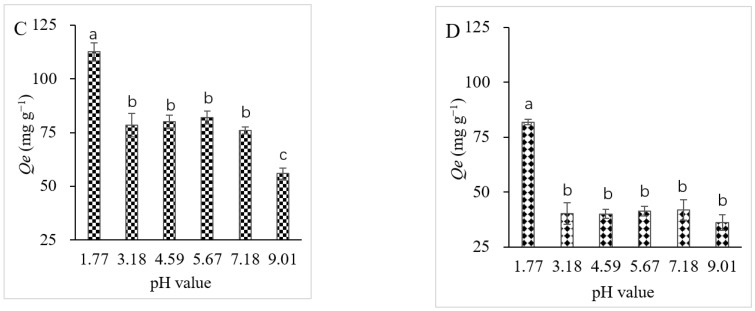
Effect of pH on adsorption of L-theanine and caffeine by ZGSPC106Na and D001SD resins. (**A**,**B**), adsorption of L-theanine by ZGSPC106Na and D001SD; (**C**,**D**), adsorption of caffeine by ZGSPC106Na and D001SD; *Qe*, adsorption capacity at equilibrium. Different letters (a–e) indicate significantly different adsorption capacities at *p* < 0.05.

The adsorption of L-theanine onto the two cation exchange resins, ZGSPC106Na and D001SD, was significantly affected by pH value (Figure 2A,B). Higher adsorption capacity was observed for both resins when acidity ranged from pH 3.18 to pH 7.18, the highest of which was witnessed at pH 5.67, and the adsorption capacities were 369.77 mg g^−1^ by ZGSPC106Na and 334.16 mg g^−1^ by D001SD, respectively. However, when the pH value was extremely low (pH = 1.77) or high (pH > 8), the adsorption capacities of L-theanine by the two resins significantly decreased and, when adsorption was performed at pH 10.13, the capacity reduced to nearly half of that at pH 5.67. This indicated that, when the pH value of the solution was close to the isoelectric point (pI) of L-theanine (pH = 5.7), the L-theanine was easy to be adsorbed onto the resins, which led to the highest adsorption capacity under this condition. The results also implied that alkaline condition was beneficial to the elution of the L-theanine. In addition, under various pH conditions, the adsorption capacities of L-theanine by ZGSPC106Na were higher than those by D001SD. 

The adsorption behaviors of caffeine by ZGSPC106Na and D001SD at different pH values were also studied. As shown in Figure 2C,D, both resins showed the highest adsorption capacities for caffeine (112.54 mg g^−1^ for ZGSPC106Na and 81.84 mg g^−1^ for D001SD) at pH 1.77. When the pH value ranged from 3.18 to 7.18, the adsorption capacities remained relatively stable (75.96–82.00 mg g^−1^ for ZGSPC106Na and 40.00–41.88 mg g^−1^ for D001SD), but significantly lower than that at pH 1.77. When acidity changed to pH 9.01, the adsorption capacities dropped remarkably. It seemed that caffeine could be easy to exchange onto the surface of the resins only at a low pH value. The comparison showed that ZGSPC106Na could adsorb much more caffeine than D001SD under the same acidity condition. Meanwhile, the adsorption capacities of ZGSPC106Na and D001SD for caffeine were much lower than that for L-theanine (Figure 2). Besides influencing the adsorption capacities, the pH value significantly affected the selectivity coefficients of both resins for L-theanine and caffeine (Table 2). Higher selectivity coefficients of the two resins for theanine against caffeine were achieved from pH 4.59 to pH 7.18, but lower coefficients were obtained at pH 1.77. Concerned with the adsorption capacity and selectivity, acidity ranging from pH 4.59 to pH 7.18 was suitable for separating the theanine from the mixture containing caffeine. Meanwhile, ZGSPC106Na resin showed better adsorption capacity than D001SD for both L-theanine and caffeine, but D001SD showed better adsorption selectivity for theanine over caffeine than ZGSPC106Na at the same pH value.

#### 3.2.2. Adsorption Kinetics

**Figure 3:**
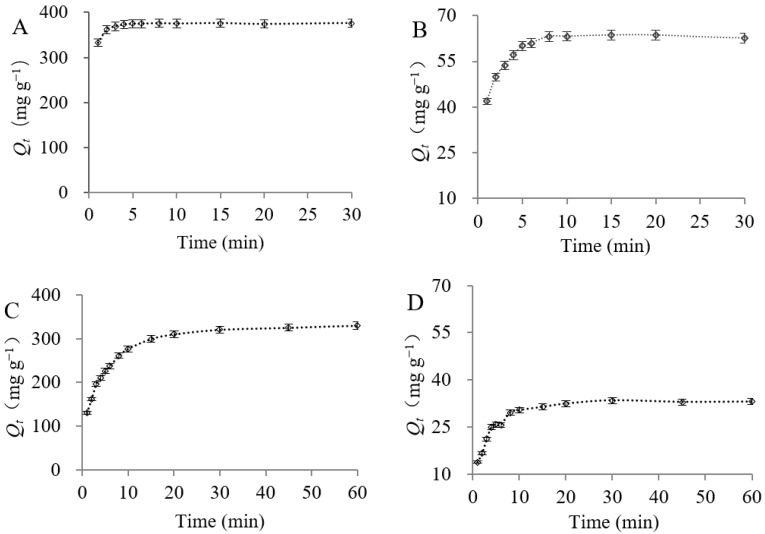
Adsorption behavior of the L-theanine and caffeine by the ZGSPC106Na and D001SD after different contact times. (**A**,**B**), adsorption of L-theanine and caffeine by ZGSPC106Na; (**C**,**D**), adsorption of L-theanine and caffeine by D001SD.

The adsorption kinetics of L-theanine and caffeine by ZGSPC106Na and D001SD were studied. As shown in Figure 3, adsorption of L-theanine onto ZGSPC106Na was performed rapidly and reached equilibrium (~370 mg g^−1^) within 5 min, but adsorption of caffeine was achieved slowly and reached equilibrium (~63 mg g^−1^) around 10min. Unlike ZGSPC106Na, D001SD required around 30 min to reach equilibrium with relatively low capacity for the L-theanine and caffeine. This indicated that not only adsorption sites for L-theanine and caffeine were relatively fewer on/in the resin D001SD, but also these adsorbates were relatively difficult to exchange with the ions of D001SD in comparison with ZGSPC106Na.

After fitting the experimental data with both the pseudo-first-order and the pseudo-second-order rate equations (Figure S1), the result showed that the adsorption of L-theanine and caffeine onto the two resins followed the pseudo-second-order model rather than the pseudo-first-order model since the correlation coefficients were all higher than 0.999 and the relative errors were less than 4% in the pseudo-second-order model (Table 3). 

It was well known that diffusion was the rate-limiting step when the adsorption process could be described by the pseudo-first-order rate equation and that the adsorption reaction was the rate-limiting step when the process could be described by the pseudo-second-order rate equation. The experimental data fitted better with the pseudo-second-order rate equation, implying that the electrostatic interaction between resins and adsorbates (L-theanine and caffeine) was the main factor affecting the adsorption rate instead of the diffusion of the adsorbates. In addition, the initial adsorption rates (*h*_2_) of the L-theanine and caffeine by ZGSPC106Na were 25.6 and 11.7 fold as high as those by D001SD, which might be the main reason for the short equilibrium time and large capacity during ZGSPC106Na adsorption. The *h*_2_ of L-theanine was 14.7 fold as high as that of caffeine by using the ZGSPC106Na as adsorbent and 6.7 fold by using D001SD as adsorbent, implying that adsorption of L-theanine was much more rapid than that of caffeine by both adsorbents, which might be related to much more net charge but a simpler structure of theanine in comparison with caffeine.

#### 3.2.3. Adsorption Isotherm

**Figure 4:**
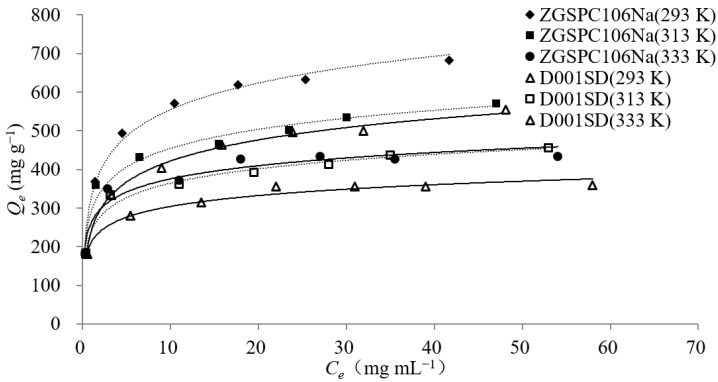
Isothermal adsorption of L-theanine onto ZGSPC106Na and D001SD.

The effect of temperature and initial concentration on the adsorption of L-theanine onto ZGSPC106Na and D001SD was studied. As shown in Figure 4, for both resins, ZGSPC106Na and D001SD, the adsorption capacities of L-theanine increased with an increase in equilibrium concentration of the adsorbate and decreased as temperature increased. This indicated that L-theanine adsorption by both resins was a spontaneously exothermic process.

Langmuir and Freundlich models were employed to fit the isothermal adsorption behaviors. The result showed that both models could be used to describe the isothermal adsorption behavior of the L-theanine onto the two resins since the correlation coefficients of the equations were all higher than 0.97 (Table 4). According to the Langmuir model, the maximum monolayer adsorption capacity (*Q_m_*) of L-theanine by ZGSPC106Na (from 609.59 mg g^−1^ to 420.33 mg g^−1^) and D001SD (from 481.61 mg g^−1^ to 345.10 mg g^−1^) decreased significantly with an increase in temperature from 293 K to 333 K, while the affinity constant (*k_L_*) of L-theanine increased along with an increase in temperature. This could be explained by the fact that high temperature not only promoted the adsorption of L-theanine, but also accelerated the desorption process. When the promotion of desorption at a high temperature exceeded adsorption, a decrease in *Q_m_* was observed. According to the Freundlich model, both the Freundlich constant (*K_F_*) and the heterogeneous factor (*1/n*) decreased with the increase in temperature. It was clear that high temperature would accelerate the molecule movement and lead to promote the entrance of adsorbates into adsorption sites, but to decrease the interaction between the adsorbent and the adsorbate, which in turn caused a decline in adsorption capacities.

The comparison also showed that the correlation coefficients of the Langmuir equation during adsorption of L-theanine by ZGSPC106Na were higher than that by D001SD and the correlation coefficients of the Freundlich equation during adsorption of L-theanine by D001SD were higher than that by ZGSPC106Na. This implied that monomolecular layer adsorption mainly occurred between ZGSPC106Na and L-theanine while a mixed adsorption mode including monomolecular and multimolecular layers’ interaction might occur between D001SD and L-theanine, since the Langmuir isotherm was suitable for describing the monomolecular layer adsorption behavior and the Freundlich model was usually used to describe the behavior of the monomolecular layer adsorption, as well as the multimolecular layer adsorption [33]. 

#### 3.2.4. Elution Efficiency of the Different Eluents

After the contact of the 20 mg mL^−1^ theanine with the resins, five eluents including 1.0 mol L^−1^ NaCl solution, 0.133 mol L^−1^ Na_2_HPO_4_ solution, 0.5 and 1.0 mol L^−1^ ammonia solution, 1.0 mol L^−1^ Na_2_CO_3_ solution were tried to elute the theanine from the adsorbents. For ZGSPC106Na, more than 90% of adsorbed theanine could be eluted by eluent Na_2_HPO_4_, around 80% by 1.0 mol L^−1^ ammonia solution and around 70% by NaCl and Na_2_CO_3_, only 35% by 0.5 mol L^−1^ ammonia solution (Table 5). For D001SD, a similar but high elution rate was observed by these eluents; in particular, almost all of the adsorbed theanine could be eluted by Na_2_HPO_4_ and more than 90% by 1 mol L^−1^ ammonia solution. It was clear that elution efficiency was influenced by the concentration of cations as well as buffer capacity, and L-theanine could be easily eluted from D001SD instead of ZGSPC106Na. Considering the easy removal of NH_3_ from eluates, the 1 mol L^−1^ ammonia solution was recommended as the preferred eluent although its elution efficiency was slightly lower than 0.133 mol L^−1^ Na_2_HPO_4_. 

### 3.3. Simultaneous Preparation of Tea Components

Three powders (P_A_, P_B_ and P_C_) were achieved after the green tea extract was successively separated by PA-6, HPD-400 and D001SD columns according to the simultaneous preparation procedure as described in the materials and methods. The content of catechins was 854.08 mg g^−1^ (total polyphenols 99.1%) with a recovery rate of 74.48% in the powder P_A_ which was recovered from the PA-6 column at the 80% ethanol elution stage. Powder P_A_ was contaminated with very few L-theanine (4.63 mg g^−1^) and caffeine (2.53 mg g^−1^). The result indicated that catechins could be effectively enriched after being adsorbed by a PA-6 fixed bed just once. In powder P_B_ obtained from the HPD-400 column at the 80% ethanol elution stage, the content of caffeine was 502.61 mg g^−1^ with a recovery rate of 69.65%; a certain amount of catechins (299.31 mg g^−1^) but very little L-theanine (2.53 mg g^−1^) were detected. It was clear that the caffeine could be enriched by the HPD-400. After treatment, impurities were removed after elution by water and ethanol. The content of L-theanine in powder P_C_ obtained from the D001SD column at the ammonia elution stage, was 373.30 mg g^−1^ (total amino acids 621.12 mg g^−1^) with a recovery rate of 71.04% and very few catechins (1.66 mg g^−1^) and caffeine (1.95 mg g^−1^) contamination. This indicated that L-theanine could be largely purified by D001SD adsorption.

## 4. Discussion

### 4.1. Adsorbent Properties Determine the Adsorption Behavior

In the present study, the adsorption behaviors of L-theanine by cation exchange resins ZGSPC106Na and D001SD were studied. ZGSPC106Na showed higher adsorption capacities than D001SD for both L-theanine and caffeine, while D001SD showed better selectivity for L-theanine over caffeine (Figure 1, Table 1 and Table 2). According to the product description, these two resins belong to different types although they possess similar styrene skeletons and functional groups (–SO_3_H). The ZGSPC106Na is a gel-type resin (www.chinaresin.com (accessed on 1 January, 2020)), while the D001SD is a macro-porous type of resin (www.amicogenchina.cn (accessed on 1 January, 2019)). The total exchange capacity of the former (≧4.50 mmol/g) is bigger than that of the latter (≧4.35 mmol/g), which might be a reason for a relatively higher adsorption capacity of L-theanine and caffeine by the former instead of the latter. The different adsorption selectivity for L-theanine over caffeine might relate to the distinctive structure of these two resins, since the D001SD possesses a larger number of real micropores in the matrix which are attributed to the effect of porogen during the synthesis process and lead to the different mass transfer selectivity, whilst the ZGSPC106Na only bears the spaces (but not the real micropores) between crosslinking and hydrated polymers which do not actually exist in dry resin. Moreover, the different time of adsorption equilibrium also reflected the structure distinction between the two resins. According to the test of adsorption kinetics, the D001SD possesses a lower adsorption capacity for L-theanine and caffeine than ZGSPC106Na, but needs a longer time to reach adsorption equilibrium (Figure 3, Table 3). Compared with the opened homogeneous space of ZGSPC106Na, micropores of D001SD exhibit much more diversity and limited entrance, which certainly will increase the diffusion difficulty and prolong the equilibrium duration of the adsorbates. In addition, adsorption of L-theanine could be well fitted to the Langmuir and Freundlich models, indicating that the adsorption behavior of L-theanine onto the two resins was influenced significantly by the concentration of adsorbates in solution and the unoccupied sites on/in adsorbent [34]. The adsorption behavior was confirmed as a spontaneous and exothermic process, and higher initial concentration and lower temperature would benefit in achieving a higher adsorption capacity. Maximum L-theanine could be adsorbed at 293 K by ZGSPC106Na (609.59 mg g^−1^) and D001SD (481.61 mg g^−1^), which was higher than other reported adsorbents [21]. Interestingly, according to the fitness calculation of the two models, the Langmuir model was much more suitable for describing the adsorption of L-theanine onto the ZGSPC106Na, while the Freundlich model was more appropriate for describing the adsorption of L-theanine onto the D001SD. This was in line with the structure difference between the two resins. As a gel-type resin, the ZGSPC106Na has more homogeneous internal spaces after hydration and all the functional groups could be regarded as monomolecular layer adsorption sites with similar characteristics. As a macro-porous type of resin, the functional groups of the D001SD might be largely considered as multimolecular layer sites since the diameter and depth of micropores were usually heterogeneous. In view of the better selectivity, reusability, pollution resistance and higher mechanical strength of macro-porous resin, D001SD was recommended to be used to separate the L-theanine from a complex matrix, although its adsorption capacity was lower than gel resin ZGSPC106Na.

### 4.2. Ionization Influences the Adsorption and Elution

Acidity conditions affected not only adsorption capacity but also selectivity, mainly through impacting on the ionization of the adsorbents and the adsorbates. When the acidity of the adsorption solution was close to pH 5.7, the net charge of L-theanine was close to 0 [35], but the functional group of the two resins still could easily ionize as –SO_3_^−^ [36]. Under such conditions, L-theanine possessed the lowest ionization level and activity in the solution and was prone to move into the micropores or spaces of the resins and be arrested onto the ionized functional group –SO_3_^−^ through electrostatic interaction. When the acidity of the solution increased extremely to around pH 1.75, although the –SO_3_H group was a strong acid, increased difficulty in ionization of the group would lead to a remarkable decrease of the adsorption sites; meanwhile, the L-theanine bore much more net positive charges, which would limit its diffusion in the micropore or space of the resins. Considering the combination of the two facts, the adsorption capacity of the resins for L-theanine decreased sharply (Figure 2). When the solution pH value increased up to pH 9.0, the –SO_3_H fully ionized, but the L-theanine possessed more net negative charges which would disturb its arrest onto the functional group of the resins. As a result, the adsorption capacity of L-theanine also decreased under high pH values. It can be seen that the effect of pH value on the adsorption capacity of L-theanine is not only related to the net charge of the adsorbates, but also the adsorption site of the adsorbents. After comparing the adsorption capacity of caffeine under different acidity conditions, significantly higher capacity was only observed at extremely low pH (~pH 1.75), mainly due to the fact that caffeine is difficult to ionize under normal acidity and can bear much positive charge only at very low pH (Figure 2). Low charge will make caffeine difficult to be arrested by the functional group of the resins, which leads to relatively lower adsorption capacity of caffeine in comparison with L-theanine. From our experimental results (Figure 1 and Figure 2), mainly through the ion exchange and electrostatic interaction, caffeine was adsorbed onto the resin through the Van der Waals force or hydrophobic interaction with the backbone of the polymers since caffeine possessed strong hydrophobicity with a perfectly conjugated electron cloud [37]. This might also be the key reason why ZGSPC106Na and D001SD resins could adsorb relatively a high level of catechins (Figure 1). In addition, different elution efficiency of the various eluents might also reflect the effect of pH value and ionization on desorption. An eluent with strong ionization and buffer capacity, such as 0.133 mol L^−1^ Na_2_HPO_4_, could thoroughly elute the adsorbates from adsorbents. According to our results, adsorption under weakly acidic conditions and desorption under basic conditions were suitable for the separation of the L-theanine.

### 4.3. Comprehensive Preparation Technology for Multiple Targets

Promotion of the target concentration and removal of the impurity are the most important technology for preparing the tea components. Many attempts have been made to separate the different components of tea, such as catechins, theanine and polysaccharides. After extraction, decaffeination is a key purification step for obtaining the target components. Decaffeinated catechins could be achieved by organic solvent partition, such as with ethyl acetate [38]. Solvent residues were usually unavoidable, which limited the product application, especially in functional foods and pharmacies. Liang et al. [39] established an efficient method to remove the caffeine from fresh leaves through treatment with hot water in a short time, by which 83% of caffeine was removed and 95% of total catechins were retained. Unfortunately, this approach was only suitable for the decaffeination of fresh tea leaves. Decaffeination through supercritical carbon dioxide (SC-CO_2_) extraction was quite secure because of low solvent residue, but loss of catechins seemed unavoidable when co-solvent was used to improve the efficiency of caffeine removal; in particular, loss of ~20% catechins [40] and 21–37.8% of the initial EGCG [41,42] was observed when using water as the co-solvent and loss of 41–68% catechins when using ethanol as co-solvent [43]. When ethyl lactate was used as a co-solvent, decaffeinated green tea extract with high catechins (23%) and low caffeine (<1%) could be produced through SC-CO_2_ extraction [44,45]. It was clear that further study needed to be conducted to clarify the effect of materials or the role of co-solvent. Poly (acrylamide-co-ethylene glycol dimeth-acrylate) [25], polyamide [24], PVPP [23], lignocellulose [46] and chemically modified lignocellulose [47] were used as adsorbates to separate the catechins from a complex matrix and highly purified catechins could be achieved after elution with gradient ethanol. Our study confirmed that polyamide PA-6 was an efficient adsorbent for preparing the catechins from tea extract because of its high adsorption capacity and selectivity. 

Ion exchange resins were usually employed to isolate L-theanine from tea extract, since L-theanine was an amphoteric compound with free amino and carboxy groups. Ye et al. [21] studied the adsorption behavior of L-theanine by cation exchange resin No.732 and obtained highly purified L-theanine at a recovery rate of 84.96%. It was not clear whether the resin No.732 was suitable for preparing high purity theanine directly from tea extract, since the initial material was a partially purified crude L-theanine, but not an original green tea extract in this report. Zhang et al. [19] established a theanine preparation method in which L-theanine was partially purified with 50% purity after separating the theanine through a column chromatograph fulfilled with No.732 cation exchange resin and highly purified (98%) with a recovery rate of 70.4% by a subsequent preparative HPLC. However, this technology was only suitable for small-scale preparation in the lab. Molecularly imprinted polymer (MIP) was also attempted to isolate theanine from green tea extract [18], but this approach needed to be further optimized for promotion of the loading capacity. Therefore, it is not feasible to directly prepare theanine from tea infusion, especially on a large scale, since the abundance of theanine in tea is usually lower than 1.5%, whilst it is of practical significance to recover the theanine from the by-product of tea polyphenols preparation. 

**Figure 5:**
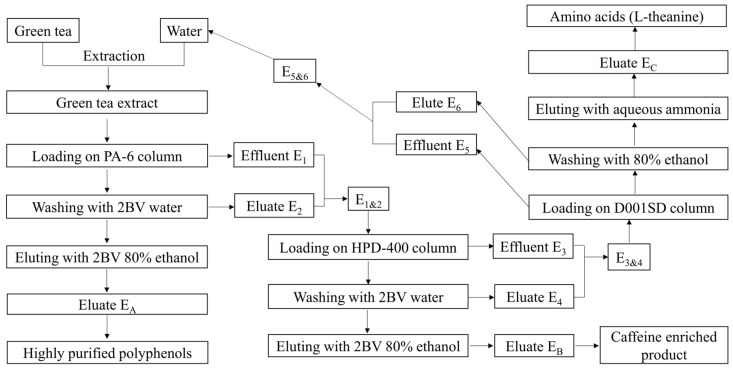
Simultaneous preparation of polyphenols, L-theanine and caffeine from green tea through column separation.

Simultaneous preparation would contribute to the comprehensive utilization of the tea bioactive compounds and cost reduction. Polyphenols, theanine and caffeine could be simultaneously achieved from green tea extract at purities of 96.27% (recovery rate 72.42%), 99.02% (recovery rate 66.12%) and 99.25% (recovery rate 62.07%), respectively, through separation with a polyamide column and a macro-porous resin NKA-Ⅱ column [48]. However, a complex elution program and a large elution volume were used, which made it impossible to apply in large-scale production. In our study, based on the comprehensive consideration of product purity, solvent volume, time consumption and large-scale application, the green tea extracts were successively subjected to separation with PA-6, HPD-400 and D001SD columns, during which a two-step elution was conducted for each adsorbent (Figure 5). Three types of products could be achieved, including decaffeinated polyphenols (catechins), an extract with a high content of caffeine, as well as decaffeinated amino acids (L-theanine). In detail, highly purified polyphenols with a purity of 99.0% and a recovery rate of 86.77% were obtained at the ethanol elution stage of PA-6 adsorption, in which the content of catechins was higher than 85%, while contaminated caffeine and theanine were both less than 0.5%. Decaffeinated amino acids (>62%) with very little contamination of catechins (<0.2%) and caffeine (<0.2%) were achieved at the aqueous ammonia elution stage of DS001D adsorption, in which the purity of L-theanine was around 37% with a recovery rate of 71.04%, indicating that a substantial amount (~38%) of unknown compounds were present. These unknown substances might include inorganic salts and proteins according to the phenomenon that they too could be adsorbed by the DS001D. Therefore, the prepared amino acid (L-theanine) could be directly applied in food additives or functional drinks because it was almost free of caffeine and catechins. According to our study, the purity of L-theanine could be elevated during D001SD column separation by elution with gradient aqueous ammonia which would provide differential ion strength and elution ability, but this operation inevitably increases the complexity of the elution procedure. Meanwhile, the L-theanine could be further purified through methanol recrystallization in which L-theanine saturated solution was prepared by using aqueous methanol as solvent at a high temperature (60 °C), followed by filtration to remove impurities at room temperature and crystallization of theanine at a low temperature (4 °C). After the recrystallization treatment, the purity of L-theanine could exceed 98% [22]. In addition, in order to improve the recovery rate, the collected effluent (E_5_) at loading stage and eluate (E_6_) at 80% ethanol elution stage during D001SD separation could be pooled and evaporated for ethanol removal and then used as an initial solvent to brew the tea for another batch preparation (Figure 5).

## 5. Conclusions

Separation of various components from green tea was studied through the adsorption of different adsorbents. According to the analysis of the adsorption capacity and selectivity, PVPP and PA-6 were suitable for separation of catechins, macroporous resin HPD-400 for caffeine, and cation exchange resin ZGSPC106Na and D001SD for L-theanine. The adsorption behavior of L-theanine and caffeine onto cation exchange resins was significantly affected by the pH value; adsorption under weakly acidic condition and desorption under basic condition were suitable for L-theanine separation. ZGSPC106Na adsorbed a higher level of L-theanine, but D001SD exhibited higher selectivity coefficient for L-theanine against caffeine. These were attributed to the distinctive structure of the two cation resins and different ionization of the adsorbates. The adsorption of L-theanine onto the cation resins could be described by the pseudo-second-order rate equation and fitted to Langmuir and Freundlich models. The maximum adsorption capacity of L-theanine decreased significantly with increase in temperature. A method for simultaneous preparation of tea components was established through successive column separation, in which decaffeinated polyphenols (catechins), extract enriched in caffeine and decaffeinated amino acids (L-theanine) were recovered from PA-6, HPD-400, D001SD column, respectively. The method lays a foundation for the further application of these components. 

## Figures and Tables

**Table 1:** Selectivity coefficients of various resins in a solution prepared from tea extract with a high level of theanine.

Resins	$K_{Caf}^{Thea}$	$K_{TCs}^{Thea}$	$K_{Caf}^{TCs}$	$K_{Thea}^{TCs}$	$K_{Thea}^{Caf}$
ZGSPC106Na	5.63 ± 0.32 a	1.73 ± 0.06 c	3.25 ± 0.18 b	-	-
D001SD	10.66 ± 1.50 a	3.43 ± 0.53 b	3.11 ± 0.44 b	-	-
PVPP	-	-	21.17 ± 0.67 b	38.73 ± 0.85 a	0.19 ± 0.02 c
PA-6	-	-	5.39 ± 0.38 b	27.65 ± 1.27 a	1.46 ± 0.04 c
HPD-400	-	-	0.18 ± 0.02 c	22.54 ± 1.15 b	42.82 ± 1.57 a

The pretreated adsorbents (equivalent to 0.500 g dry weight for each) were mixed with 25.0 mL adsorbate solution (30 mg mL^−1^ tea extract with a high level of L-theanine), respectively. Each mixture was shaken in the HZ-9201K constant temperature oscillator (Taicang, China) at 25 °C and 150 rpm for 12 h. Thea. L-theanine; Caf. caffeine, TCs. total catechins. Different letters in a row indicated a significant difference at *p* < 0.05.

**Table 2:** Selectivity coefficient $K_{Caf}^{Thea}$ under different pH conditions.

Resins	pH 1.77	pH 3.18	pH 4.59	pH 5.67	pH 7.18	pH 9.01
ZGSPC106Na	1.46 ± 0.07 d	9.18 ± 0.42 c	12.80 ± 0.65 a	12.39 ± 0.92 ab	13.71 ± 0.60 a	11.10 ± 0.47 b
D001SD	1.75 ± 0.08 e	12.68 ± 0.68 d	14.18 ± 1.09 bcd	16.85 ± 1.23 a	15.99 ± 1.22 ab	13.31 ± 0.90 cd

Initial concentrations of L-theanine and caffeine were 20 mg mL^−1^ and 10 mg mL^−1^, respectively; $K_{Caf}^{Thea}$ indicated the selective adsorption of theanine (Thea) against caffeine (Caf). Different letters in a row indicated a significant difference at *p* < 0.05.

**Table 3:** Adsorption kinetic parameters of L-theanine and caffeine onto cation resins.

	ZGSPC106Na		D001SD	
	Theanine	Caffeine	Theanine	Caffeine
*Q_e_*_.exp_ (mg g^−1^)	376.1475	63.5902	332.1596	33.2479
Pseudo-first-order model				
*Q_e_*_.cal_ (mg g^−1^)	26.0421	0.7375	139.8328	10.8186
*h*_1_ (mg g^−1^ min^−1^)	5.8685	0.2246	9.1735	0.4322
*k*_1_ (min^−1^)	0.2253	0.3045	0.0656	0.0399
Relative error (%)	93.0766	98.8402	57.9019	67.4608
R	0.8358	0.9807	0.9681	0.8238
Pseudo-second-order model				
*Q_e_*_.cal_ (mg g^−1^)	376.7253	63.0695	342.4335	34.1917
*h*_2_ (mg g^−1^ min^−1^)	3850.8837	261.7727	150.5541	22.4027
*k*_2_ (g mg^−1^ min^−1^)	0.0271	0.0658	0.0013	0.0192
Relative error (%)	0.1536	0.8188	3.0931	2.8387
R	0.9999	0.9999	0.9999	0.9997

Initial concentrations of L-theanine and caffeine were 20 mg mL^−1^ and 10 mg mL^−1^, respectively. *Q_e_*_.exp_ represented the measured value through experiment, while *Q_e_*_.cal_ represented the theoretical value calculated from Equations (3) and (4). *h*_1_ = *k*_1_ × *Q_e_*_.cal_, *h*_2_ = *k*_2_ × *Q_e.cal_*^2^, Relative error = 100 × (|*Q_e.cal_* − *Q_e_*_.exp_|)/*Q_e.exp_*_._ R was the correlation coefficient of the equation.

**Table 4:** Isothermal adsorption parameters of the L-theanine by the ZGSPC106Na and D001SD, according to Langmuir and Freundlich models.

Resins	Temperature (K)	Langmuir Isotherm				Freundlich Isotherm			
		Model	*k_L_* (mL mg^−1^)	*Q_m_* (mg g^−1^)	R	Model	*1/n*	*k_F_* (mg^(*n*−1)/*n*^ g^−1^ mL ^1/*n*^)	R
ZGSPC106Na	293	1/*Q_e_* = 0.0011/*C_e_* + 0.0016	1.49	609.59	0.9960	*lnQ_e_* = 5.69 + 0.25 *lnC_e_*	0.25	296.39	0.9850
	313	1/*Q_e_* = 0.0010/*C_e_* + 0.0020	1.89	508.09	0.9966	*lnQ_e_* = 5.60 + 0.21 *lnC_e_*	0.21	270.73	0.9802
	333	1/*Q_e_* = 0.0010/*C_e_* + 0.0024	2.47	420.33	0.9968	*lnQ_e_* = 5.51 + 0.17 *lnC_e_*	0.17	246.24	0.9745
D001SD	293	1/*Q_e_* = 0.0016/*C_e_* + 0.0021	1.28	481.61	0.9906	*lnQ_e_* = 5.45 + 0.23 *lnC_e_*	0.24	232.50	0.9949
	313	1/*Q_e_* = 0.0014/*C_e_* + 0.0024	1.75	414.90	0.9944	*lnQ_e_* = 5.44 + 0.18 *lnC_e_*	0.18	231.33	0.9849
	333	1/*Q_e_* = 0.0014/*C_e_* + 0.0030	2.03	345.10	0.9882	*lnQ_e_* = 5.33 + 0.15 *lnC_e_*	0.15	207.21	0.9908

*k_L_*. affinity constant of Langmuir model, *Q_m_*. maximum monolayer adsorption, R. correlation coefficient; *1/n*. heterogeneous factor, *k_F_*. Freundlich constant.

**Table 5:** Elution efficiency of L-theanine by different eluents.

Resins	Adsorption Amount (mg g^−1^)	Elution Rate (%)				
		1 mol L^−1^ NaCl	0.133 mol L^−1^ Na_2_HPO_4_	1 mol L^−1^ Na_2_CO_3_	0.5 mol L^−1^ NH_3_·H_2_O	1 mol L^−1^ NH_3_·H_2_O
ZGSPC106Na	366.72 ± 3.41	72.07 ± 0.67 c	92.67 ± 0.86 a	70.45 ± 0.66 c	34.68 ± 0.16 d	79.46 ± 1.16 b
D001SD	329.88 ± 3.60	80.54 ± 0.88 c	98.58 ± 1.08 a	78.71 ± 0.86 c	47.09 ± 0.30 d	93.63 ± 2.39 b

Elution rate was calculated according to Equation (7). Different letters in a row indicate significant difference at *p* < 0.05.

## Data Availability

Data is contained within the article.

## Supplementary Materials

The following supporting information can be downloaded at: https://www.mdpi.com/article/10.3390/foods11223625/s1, Figure S1: Fitness of the pseudo-second-order model for the adsorption of L-theanine(A) and caffeine (B) onto cation exchange resin.
