# Supplementary material for: Adsorption Behavior of the L-Theanine onto Cation Exchange Resin ZGSPC106Na and D001SD
Source: Foods. 2022 Nov 13;11(22):3625. doi: 10.3390/foods11223625 (PMC9689365; doi:10.3390/foods11223625)
Supplement: Supplementary file 1 [file foods-11-03625-s001.zip › foods-2005149-supplementary.pdf]

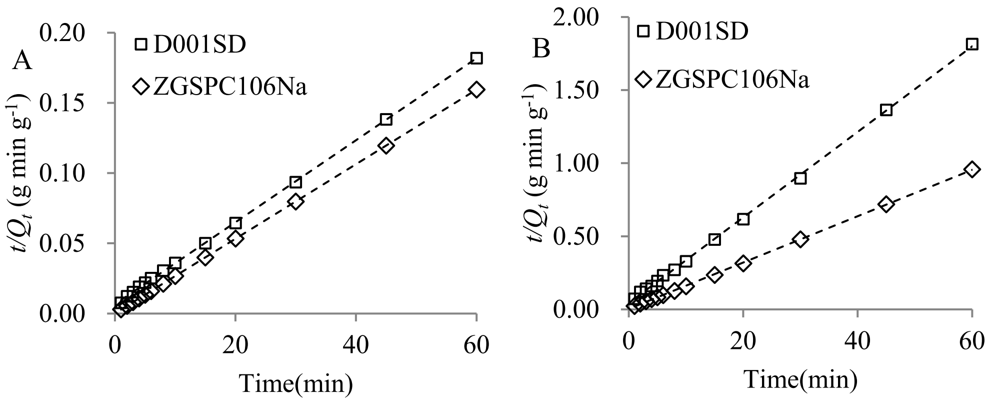

Figure S1. Fitness of the pseudo-second-order model for the adsorption of L-theanine(A) and caffeine (B) onto cation exchange resin.
